# Long-Term γ-Hydroxybutyric Acid (GHB) and Disulfiram Combination Therapy in GHB Treatment-Resistant Chronic Alcoholics

**DOI:** 10.3390/ijerph8072816

**Published:** 2011-07-06

**Authors:** Angelo Giovanni Icro Maremmani, Pier Paolo Pani, Luca Rovai, Matteo Pacini, Liliana Dell’Osso, Icro Maremmani

**Affiliations:** 1 Vincent P. Dole Dual Diagnosis Unit, Santa Chiara University Hospital, Department of Psychiatry, NPB, University of Pisa, Via Roma, 67 56100 Pisa, Italy; E-Mails: angelogimaremmani@gmail.com (A.G.I.M.); lucarovai@yahoo.com (L.R.); ldelloss@psico.med.unipi.it (L.D.); 2 AU-CNS, “From Science to Public Policy” Association, Pietrasanta, Lucca, 55045, Italy; 3 Social-Health Direction, Health District 8 (ASL 8) Cagliari, 09100 Italy; E-Mail: pallolo@tin.it; 4 G. De Lisio Institute of Behavioral Sciences, Pisa, 56100, Italy; E-Mail: paciland@virgilio.it

**Keywords:** GHB-disulfiram combination, alcohol dependence, long-term treatment

## Abstract

Leading Italian studies support the use of γ-hydroxybutyric acid (GHB), not only in the treatment of the alcohol withdrawal syndrome, but also in maintaining alcohol abstinence. GHB gives a better result than naltrexone and disulfiram in maintaining abstinence, and it has a better effect on craving than placebo or disulfiram. The problem is that about 30–40% of alcoholics are non-responders to GHB therapy. In our clinical practice, we speculate that by combining disulfiram with GHB treatment we may be able to achieve a kind of ‘antagonist’ effect by using the ‘psychological threat’ of disulfiram (adversative effect) while taking advantage of the anticraving effect of GHB, despite the limitation of its ‘non-blockade’ effect on alcohol. In this context, to improve the outcome in GHB long-term treated alcoholics, we added disulfiram to GHB in the management of GHB treatment-resistant alcoholics. In this study we compared retention in treatment of 52 patients who were treated with the GHB-disulfiram combination for up to six months, with retention for the same subjects considering their most recent unsuccessful outpatient long-term treatment with GHB only. An additional comparison was carried out on the days of complete abstention from alcohol. Thirty four patients (65.4%) successfully completed the protocol and were considered to be responders; 18 (34.6%) left the programme, and were considered to be non-responders. Considering the days of complete abstinence from alcohol, 36 patients stayed in treatment longer with the GHB-Disulfiram combination, 12 stayed for a shorter time and four for the same time. The results of this study seem to indicate a higher efficacy of the GHB-disulfiram association compared with GHB alone. Randomized controlled trials are now needed to verify this hypothesis.

## 1. Introduction

Alcohol abuse and alcoholism are a world-wide problem, from a medical but also a social viewpoint. In the past the therapy for alcoholics was mainly based on the psychosocial approach, but the combined use of pharmacotherapy and psychosocial interventions has raised the percentage rate of success in maintaining alcoholic patients in remission [1,2]. Currently there are only three medications that have been approved by the U.S. Food and Drug Administration (FDA) for use in treating alcohol abuse and alcoholism: disulfiram [3] naltrexone [4–6] and acamprosate [7,8]. Leading Italian studies also support the use of γ-hydroxybutyric acid (GHB). In alcohol dependence, in fact, one of the neurotransmitter systems most directly involved is the GABA-ergic one. γ-Aminobutyric acid (GABA) is the major inhibitory neurotransmitter in the central nervous system and this explains why GABA-ergic medications, such as GHB, are promising drugs with potential uses in treating alcohol withdrawal syndrome (AWS), but also in maintaining alcohol abstinence [9]. Since 1992, GHB has been approved in Italy and Austria as a treatment for alcohol dependence [10]. Many studies have focused on the efficacy of GHB in maintaining abstinence from alcohol [11–17]. The Cochrane Collaborative Review Group showed that GHB is effective in preventing relapses in previously detoxified alcoholics at a 3-month follow-up. The side-effects of GHB do not differ statistically from those induced by benzodiazepines, naltrexone or disulfiram [18]. A key drawback is that about 30–40% of alcoholic subjects are non-responders to GHB therapy [15]. In our clinical experience, the reduction, whether temporary or continuing, in the craving experienced by these patients was insufficient to control their dependence on alcohol. This could be related to the short half-life of GHB [19], so that it could be useful to divide up GHB administration into six doses per day [15,20]. Nevertheless, because of this last property some people may show a tendency to abuse GHB [21].

For over 55 years, disulfiram has been approved by the FDA for the treatment of alcohol dependence. It is a unique medication that relies on a ‘psychological threat’ to avoid disulfiram-ethanol reactions; in fact, it irreversibly inhibits aldehyde dehydrogenase (the enzyme that converts the relatively toxic metabolite acetaldehyde into the benign metabolite acetate), which is necessary to the metabolism of ethanol [22,23]. Consuming alcohol after taking disulfiram results in symptoms such as palpitations, flushing, nausea, vomiting, and headaches [24]. An evidence report from the Agency for Healthcare Research and Quality [3] concluded that four placebo-controlled randomized controlled trials (RCTs) using oral disulfiram produced mixed results. In two trials, oral disulfiram was shown to reduce the frequency of drinking days, but it failed to improve relapse rates compared with placebo [24]. Patients using disulfiram tended to remain in treatment longer than those who did not use disulfiram or used a naltrexone-based treatment [25,26]. This finding is consistent with other studies showing that disulfiram, in particular, helped patients to maintain abstinence and to stay in treatment for longer periods [27]. Much research has been carried out to demonstrate the efficacy of supervised low dose disulfiram as an effective treatment for alcoholism [22,28–30]. In some cases, disulfiram may be an effective and well-tolerated pharmacological treatment, within the framework of a well-integrated pharmacological, psychosocial and behavioural treatment programme [31]. Recently it has won attention as an agent adjunctive to other pharmacological medications, that specifically reduce alcohol craving [22]. Furthermore, the option of combining disulfiram with other anti-craving agents should be considered [32,33].

We know that an effective treatment of addiction is based on two important pharmacological concepts: (i) the drug used has to prove an ‘antagonist’ effect against the abused substance; and (ii) an ‘anti-craving’ effect against drug-seeking behaviour, by stimulating the substance of the abuse-related affected system [34]. These two concepts stand as the basis of methadone treatment [35]. GHB and disulfiram do not possess these two properties. GHB cannot be classified as an ‘antagonist’ drug, even if it displays some anticraving effect. Disulfiram is an ‘adversative drug’ that induces negative reinforcement, but it does not show any ‘anti-craving’ effect.

We speculate that, by combining disulfiram and GHB treatment, we can achieve a kind of ‘antagonist’ effect by using the ‘psychological threat’ of disulfiram (aversive effect), while taking advantage of the anticraving effect of GHB. In our clinical practice, we added disulfiram to GHB in the management of GHB treatment-resistant alcoholics.

The aim of the present study was to retrospectively evaluate the efficacy of a long-term (six-month) GHB-disulfiram combination treatment with reference to the ethanol intake of chronic alcoholics who met the criteria for GHB treatment resistance. We also studied endpoint clinical differences between responders and non-responder patients treated with the GHB-disulfiram combination. Lastly, we explored predictors of response to the GHB-disulfiram combination using patients‘ demographic and clinical characteristics at baseline.

To do that we compared the retention in treatment of patients treated with the GHB-disulfiram combination, for up to six months, to retention for the same subjects considering their most recent unsuccessful outpatient long-term treatment with GHB only. An additional comparison was carried out by comparing the days of complete abstention from alcohol in those two cases.

## 2. Methods

### 2.1. Sample

We considered all consecutive chronic alcoholics in treatment with GHB at the Dual Diagnosis Unit of the Department of Psychiatry of the University of Pisa, Italy, during a three-year period (2007–2010).

The inclusion criteria were:

- Diagnosis of alcohol dependence according to DSM-IV-R criteria [36], whether complicated or not by psychiatric comorbidity
- GHB treatment resistance, as determined by at least two periods of GHB long-term treatment in the previous two years. Patients were treated with our standard therapeutic protocol for alcohol dependence (GHB long-term treatment) and for psychiatric comorbidity, when present (use of antidepressants, generally SSRIs or bupropion, mood stabilizers, and occasionally benzodiazepines). Our GHB long-term treatment consists of three consecutive phases: (i) cessation of ethanol consumption within the first six days of treatment; (ii) progressive adjustment of the dose on the basis of the patient's response; (iii) follow-up, usually up to one year, with some patients continuing at the optimized dose, while others voluntarily suspended the treatment.
- Relapse into alcohol abuse during the current GHB long-term treatment
- Presence of multiple psychosocial or environmental problems within the past two years. Scores for social functioning when relapsing were used as the baseline ratings for the study
- Patients relapsing into the use of over five units of alcohol per day
- Patients living with their families

Exclusion criteria were:

- Serious liver disorders and chronic diseases
- Any structured psychotherapy during the last year

The sample consisted of 52 patients (23 males), mean age 40 ± 11. Patients were mostly females (55.8%), married (76.9%), unemployed (48.1%), with a low educational level (67.3%), with adequate economic resources (90.4%), but experiencing difficulties as to social adjustment. Most of them (n = 39) showed one or more psychiatric comorbidities.

### 2.2. Assessment

Alcohol intake was evaluated in terms of units of alcohol. The easiest way to calculate this is to count the number of glasses of alcoholic drinks daily. A glass of wine (which is usually 125 mL), beer (which is usually 330 mL), or a glass of whisky (which is usually 40 mL) contain the same amount of alcohol equal to about 12 grams, which is identified as one unit.

The cessation in alcohol intake was assessed through self-evaluation and as the lack of side-effects due to alcohol disulfiram intake. These outcomes were confirmed by family observer evaluation. One or two members of the family were responsible for detecting the intake of two medications (GHB and disulfiram) and patient alcohol intake. Positive outcome in the GHB-disulfiram combination treatment consisted of complete alcohol abstinence and improved social adjustment (responder patients). A failure to achieve these criteria, a refusal to take disulfiram, a voluntary dropout from the programme and, of course, relapsing into alcohol use were evaluated as a negative outcome (non-responder patients).

Psychiatric comorbidity was separately diagnosed by two residents in psychiatry and confirmed by a senior psychiatrist (I.M.).

Severity of illness, global improvement and efficacy index were evaluated by Clinical Global Impressions (CGI) [37]. Clinical Global Impressions (CGI) consists of three global scales (items). Two of the items, Severity of Illness and Global Improvement, are rated on a 7-point scale (from normal to among the most extremely ill for the Severity of Illness and from very much improved to very much worse for Global Improvement); while the third, Efficacy Index, requires a rating of the interaction of therapeutic effectiveness and adverse reactions. Efficacy Index is an attempt to relate therapeutic effects and side-effects. Therapeutic effect is regarded as gross benefit (from 1-Unchanged or Worse to 4-Marked); side-effects as cost (from 1-None to 4-Outweighs). The index, then, is analogous to net benefit. The index is calculated by dividing the therapeutic effect score by the side-effect score.

Social adjustment was evaluated by means of the Global Assessment of Functioning [36]. The GAF reports the clinician‘s judgment on the individual‘s overall level of functioning. The maximum level (scores of 91 to 100) indicates efficient functioning over a wide range of activities. Scores between 41 and 50 point to the presence of serious psychopathological symptoms or some serious impairment in social, occupational, or school functioning. The minimum score (one to 10) indicates a persistent inability to maintain minimal personal hygiene or the presence of severe involvement in thoughts of violence or suicide. Ten levels of functioning are provided. Intermediate codes are available when appropriate.

A researcher who was not informed as to variations in subjects‘ alcohol intake administered the CGI and GAF. There was a preference for the researcher not to be informed because CGI and GAF scales are to be rated exclusively with respect to psychological, psychopathological, social and occupational functioning.

### 2.3. Procedure

The therapeutic protocol consisted of three consecutive phases. Within the first seven days of treatment, 50 mg/hg/day of GHB, divided into three doses taken every four hours, were administered to relieve alcohol withdrawal symptoms and to facilitate the cessation of ethanol consumption. In a second step, disulfiram 400 mg/day was prescribed and the dosage of GHB was progressively adjusted to a maximum of 100 mg/kg/day on the basis of the patient‘s response. Considering the short half-life of GHB [38], patients were given the daily dosage of GHB according to the following modalities: either three doses taken every four hours, or six doses taken every two and one-half hours. Patients were allowed to increase their intake by themselves (with medical approval). The third step is characterized by up to six months of follow-up treatment.

We are aware that many studies suggest lower doses of supervised disulfiram. This fact should be considered when applying the GHB-disulfiram combination to the current clinical practice.

No specific psychotherapy was provided during an unplanned schedule of visits. Generally patients were seen twice or more monthly. All patients gave their informed consent before taking any medication. As a rule, all the study participants were among those who regularly used our service. This involves an intervention where there is no change to the standard service being delivered (e.g., no randomization of service users into different groups).

In our programme patients are required to be actively involved in treatment by attending the clinic whenever that is scheduled, participating in the development of their treatment plan, working towards treatment goals, meeting with medical and case management staff, and attending groups when needed. All physicians working in the programme are psychiatrists who have been trained for at least two-years in the treatment of addictive disorders.

Recorded data were completely anonymous and it was impossible to identify participants from any resulting report.

### 2.4. Statistical Analysis

The retention rate under the GHB-disulfiram combination therapy was studied using the “life-table analysis” according to Lee-Desu statistics. First the retention rate of the entire group was compared with that during their most recent unsuccessful GHB long-term treatment. The days of complete abstinence during the current and the last unsuccessful treatment were compared by applying the Wilcoxon Matched-Pairs Signed-Ranks Test. Then differences in CGI and in GAF between responders and non-responders to GHB-disulfiram combination were tested by the Student T-Test. Lastly, predictors of the response to GHB-disulfiram combination therapy were analysed using “life table analysis”. The life-table of subgroups divided by sex (male, female), age (up to 40 years old, over 40), education (<8 years, >8 years), marital status (single, married), work (white collars, blue collars, unemployed), income (poor, adequate), baseline severity of illness (5, 6, 7 score), baseline severity of social adjustment (under the mean, over the mean), psychiatric comorbidity (absence, presence), GHB daily intake (three times, six times) were all compared. We used the statistical routines of the SPSS system.

## 3. Results and Discussion

34 patients (65.4%) successfully completed the protocol and were considered to be responders; 18 (34.6%) left the programme and were considered to be non-responders. A key result was that 34 patients (65.4%) were still under treatment after six months. Table 1 shows the retention rate under treatment.

No patients left treatment without being considered non-responders. There was a major sample attrition during the first month of treatment, and a minor one during the second and the fourth. After the fourth month of treatment, no patients left the programme. Patients under long-term treatment with the GHB-disulfiram combination stayed in treatment longer (comparison: Lee-Desu statistic 13.61, df = 1, p < 0.001) than when they were treated with GHB only. More specifically, the greatest risk of leaving the GHB only therapy during participants‘ latest unsuccessful treatment occurred during the 5th and the 3th months; the next more crucial periods were, in order, the 4th, the second and the first month. No patients were in treatment after the 5th month.

Considering the days of complete abstinence from alcohol, 36 patients stayed in treatment longer with the GHB-Disulfiram combination, 12 stayed for a shorter time and four for the same time. These variations were statistically significant according to the Wilcoxon Matched-Pairs Signed-Ranks Test (z = −5.11, 2-tailed p < 0.0001).

Table 2 shows endpoint clinical differences between responder and non-responder patients. Responders differed from non-responders as to severity of illness (lower), global improvement (higher), efficacy index (more therapeutic effectiveness, no side-effects or non-interfering side-effects), GAF score (higher).

Table 3 shows predictors of response to the GHB-Disulfiram combination treatment. Sex, age, education, marital status, job, income, baseline social impairment, psychiatric comorbidity, all failed to show any effect on the retention rate. Only GHB intake (over three times a day) and a low severity at baseline predicted a better retention rate.

According to the results obtained in the present study, patients in long-term treatment with the GHB-disulfiram combination stay in treatment longer than when they are treated with GHB alone. Moreover, the GHB-disulfiram association results in a higher percentage of days of abstinence from alcohol. Responder patients compared with non-responder ones present better clinical features; are characterized by a less severe illness at baseline and an intake of GHB over three times a day.

Given the importance of dropout as a factor conditioning compliance with therapeutic programmes in the addiction field, the increased retention in treatment observed in our study stands as strong preliminary evidence in support of the GHB-disulfiram association. Moreover, the statistically significant gain in terms of days of complete abstinence from alcohol observed in patients treated with GHB-disulfiram seems to indicate a higher efficacy of this association compared with GHB alone.

As hypothesized in the introductory section, the advantages of this association can easily be attributed to the combination of the anticraving effect of GHB with the traditional adversative effect of disulfiram. However, other mechanisms have recently been taken into consideration in explaining the efficacy of disulfiram in drug dependence. These additional mechanisms, which were initially put forward to explain the efficacy of disulfiram in the treatment of cocaine addiction [39–42], have been extended to the use of this medication in treating alcohol dependence [43].

Disulfiram might be supposed to interfere with the rewarding properties of alcohol by inhibiting the noradrenergic components of craving and of highs, and be qualified as an anticraving agent. Moreover, the decrease in noradrenergic transmission mediated by disulfiram, which dampens the stress response, could also interfere with the mechanism of relapse in response to environmental influences and stressors [44–47].

The GHB-disulfiram combination might not only have a kind of ‘antagonist’ effect related to the ‘psychological threat’ or adversative effect of disulfiram and a GHB-mediated anticraving effect, but also an antirewarding effect along with a low-level, disulfiram-mediated anticraving effect. The antirewarding, disulfiram-mediated effect might be useful too against the rewarding effect of GHB (which leads to GHB abuse). This latter effect is quite similar to that described by Caputo *et al*. regarding the GHB-naltrexone combination [48].

An alternative interpretation is that treatment success may be interpreted not only as an effect of GHB, disulfiram, or the combination of both medications, but also of psychological modes of action, e.g. motivation, self help and self management. This study was, in fact, a controlled before/after study with participants acting as their own controls; that is, they have been compared with themselves, according to whether they were being treated with GHB alone or with a combination of GHB and disulfiram. Given the time-frame which was set to include the entire duration of the two treatments and to take into account possible differences between the sociodemographic or clinical conditions in the two periods of observation, we compared these conditions at the baseline for each of the two treatments. We found no differences. Moreover, since the design did not include a separate control group, we did not actually carry out a check on the effects of time. This meant that we were unable to differentiate between changes or effects that were measured at the end of the study, but which might have occurred anyway, without any intervention, and those that were due to the intervention itself.

## 4. Conclusions

The results of this study seem to indicate a greater efficacy of the GHB-disulfiram combination compared with GHB alone. Randomized controlled trials are now needed to verify this hypothesis.

## Figures and Tables

**Table 1:** Six-month survival in treatment of 52 alcoholics treated with a GHB-disulfiram combination (current treatment) compared with later treatment with GHB only. Add a descriptive label of the table here.

Month	Number at start of month		Number leaving the programme		Cumulative % survival		Hazard rate	

	L	C	L	C	L	C	L	C
1st	52	52	5	14	0.90	0.73	0.0034	0.0104
2nd	47	38	25	2	0.42	0.69	0.0242	0.0018
3th	22	36	17	0	0.09	0.69	0.0420	0.0000
4th	5	36	3	2	0.03	0.65	0.0286	0.0019
5th	2	34	2	0	0.00	0.65	0.0667	0.0000
6th	0	34		0		0.65		0.0000

L = Later treatment, C = Current treatment; Lee-Desu statistic 13.61, df = 1, p < 0.001.

**Table 2:** Endpoint clinical differences between non-responder and responder patients.

	Non-responders N = 18	Responders N = 34		

	M ± sd	M ± sd	T	p
Severity of illness	4.33 ± 0.9	1.85 ± 0.8	9.12	0.000
Global improvement	3.83 ± 1.0	1.64 ± 0.6	7.68	0.000
Efficacy index	11.33 ± 3.1	3.94 ± 2.7	8.36	0.000
GAF score	51.66 ± 6.4	68.08 ± 10.0	−7.18	0.000

**Table 3:** Predictors of response to treatment.

Group cluster	N	N Censored (%)	Lee-Desu statistics	p
Sex				
Males	23	15 (65.2)		
Females	29	19 (65.5)	0.07	0.784
Age				
≤40 years	27	20 (74.0)		
>40 years	25	14 (56.0)	2.02	0.154
Education				
≤8 years	17	12 (70.5)		
>8 years	35	22 (62.8)	0.45	0.502
Marital status				
Single	12	10 (83.33)		
Married	40	24 (60.0)	1.97	0.164
Job				
White collars	10	5 (50.0)		
Blue collars	17	13 (76.4)		
Unemployed	25	16 (64.0)	1.52	0.467
Income				
Poor	5	2 (40.0)		
Adequate	47	32 (68.0)	0.91	0.338
Baseline severity				
CGI=5	20	17 885.0)		
CGI=6	22	10 (45.4)		
CGI=7	10	7 (70.0)	7.21	0.027
Baseline social impairment				
Under the mean	26	17 (65.3)		
Over the mean	26	17 (65.3)	0.06	0.794
Psychiatric comorbidity				
Absent	13	6 (46.1)		
Present	39	28 (71.7)	1.38	0.238
GHB intake				
3 times/day	19	4 (21.0)		
6 times day	33	30 (90.9)	24.02	0.000
